# *Herbaspirillum seropedicae* Differentially Expressed Genes in Response to Iron Availability

**DOI:** 10.3389/fmicb.2018.01430

**Published:** 2018-07-03

**Authors:** María F. Trovero, Paola Scavone, Raúl Platero, Emanuel M. de Souza, Elena Fabiano, Federico Rosconi

**Affiliations:** ^1^Departamento de Bioquímica y Genómica Microbianas, Instituto de Investigaciones Biológicas Clemente Estable, Montevideo, Uruguay; ^2^Departamento de Microbiología, Instituto de Investigaciones Biológicas Clemente Estable, Montevideo, Uruguay; ^3^Departamento de Bioquimica e Biologia Molecular, Universidade Federal do Paraná, Curitiba, Brazil

**Keywords:** iron-uptake, rice, *H. seropedicae*, TonB-dependent receptor, endophyte

## Abstract

*Herbaspirillum seropedicae* Z67 is a nitrogen-fixing endophyte that colonizes many important crops. Like in almost all organisms, vital cellular processes of this endophyte are iron dependent. In order to efficiently acquire iron to fulfill its requirements, this bacterium produces the siderophores serobactins. However, the presence in its genome of many others iron acquisition genes suggests that serobactins are not the only strategy used by *H. seropedicae* to overcome metal deficiency. The aim of this work was to identify genes and proteins differentially expressed by cells growing in low iron conditions in order to describe *H. seropedicae* response to iron limitation stress. For this purpose, and by using a transcriptomic approach, we searched and identified a set of genes up-regulated when iron was scarce. One of them, Hsero_2337, codes for a TonB-dependent transporter/transducer present in the serobactins biosynthesis genomic locus, with an unknown function. Another TonB-dependent receptor, the one encoded by Hsero_1277, and an inner membrane ferrous iron permease, coded by Hsero_2720, were also detected. By using a proteomic approach focused in membrane proteins, we identified the specific receptor for iron-serobactin internalization SbtR and two non-characterized TonB-dependent receptors (coded by genes Hsero_1277 and Hsero_3255). We constructed mutants on some of the identified genes and characterized them by *in vitro* growth, biofilm formation, and interaction with rice plants. Characterization of mutants in gene Hsero_2337 showed that the TonB-dependent receptor coded by this gene has a regulatory role in the biosynthesis of serobactins, probably by interacting with the alternative sigma factor PfrI, coded by gene Hsero_2338. Plant colonization of the mutant strains was not affected, since the mutant strain normally colonize the root and aerial part of rice plants. These results suggest that the strategies used by *H. seropedicae* to acquire iron inside plants are far more diverse than the ones characterized in this work. *In vivo* expression studies or colonization competition experiments between the different mutant strains could help us in future works to determine the relative importance of the different iron acquisition systems in the interaction of *H. seropedicae* with rice plants.

## Introduction

Bacterial endophytes are classically defined as bacteria isolated from superficially sterilized plants that colonize the internal plant tissues without causing visible damage to their hosts (Reinhold-Hurek and Hurek, 2011; Gaiero et al., 2013). These groups of strains spend at least part of their life cycle in the endosphere of the plant (Hardoim et al., 2015) and are different from the ones that colonize the plant root surface (Edwards et al., 2015). Bacterial endophytes research is important because these bacteria contribute to plant health (Berg et al., 2016; Wemheuer et al., 2017). Many endophytic isolates can promote the growth and increase the fitness of the host plants by different mechanisms such as cycling nutrients, controlling pathogens, protecting against herbivores, reducing abiotic stresses, producing phytohormones, or by biological nitrogen fixation (Rosenblueth and Martinez-Romero, 2006; Ryan et al., 2008; Abdalla and Matasyoh, 2014; Hardoim et al., 2015).

A common feature present in published endophyte genomes and metagenomes is the presence of a wide variety of iron acquisition systems (Sessitsch et al., 2012; Malfanova et al., 2013; Mitter et al., 2013). Iron is an essential metal that acts as cofactor of many enzymes and participates in several cellular processes, such as respiration, photosynthesis, or nitrogen fixation (Sandy and Butler, 2009; Ilbert and Bonnefoy, 2013; Ahmed and Holmstrom, 2014). It also has a relevant role for biofilm formation in different species (Wu and Outten, 2009; Ahmed and Holmstrom, 2014). Although it is one of the most abundant elements on the Earth crust, under physiological conditions iron availability is limited (Cornelis et al., 2011).

The most common strategy used by bacteria to circumvent this low availability is the production of siderophores (Miethke, 2013). These are low molecular weight chelators with high affinity for ferric iron that, once in complex with the metal, are recognized and actively internalized to the cell cytosol by specific receptors (Andrews et al., 2003; Chu et al., 2010). In the case of Gram-negative bacteria, specific outer membrane proteins, known as TonB-dependent receptors, are the ones involved in iron-siderophore complex internalization to the periplasm (Ferguson et al., 2007; Krewulak and Vogel, 2011). Some TonB-dependent receptors contain an extra domain (pfam07660) and are usually transcribed with an anti-sigma/sigma factor cognate pair. Besides acting as transporters, these proteins act in genetic regulation by cell-surface signaling systems and are referred as TonB-dependent transducers (Koebnik, 2005; Llamas et al., 2008).

*Herbaspirillum seropedicae* Z67 (Baldani et al., 1986) is a Gram-negative diazotrophic endophytic bacterium that belongs to the β-Proteobacteria subclass and colonizes and survives inside many important agricultural crops, such as sorghum, wheat, maize, and rice (Elbeltagy et al., 2001; James et al., 2002). Like other bacterial endophytes, the genome of *H. seropedicae* SmR1, our reference genome strain, shows a high repertoire of genes putatively involved in iron acquisition systems. This includes 28 different TonB-dependent receptors, more than 4 inner membrane internalization systems, and only 1 siderophore biosynthetic cluster, the one responsible for the production and transport of serobactins, a family of siderophores produced by *H. seropedicae* in response to iron starvation (Rosconi et al., 2013). Our previous results suggested that although endogenous serobactins confers a competitive advantage on the plant colonization, *H. seropedicae* is able to use alternative iron sources besides serobactins to deal with iron deficiency, because the serobactin defficient mutant is still able to colonize and survive inside rice plants (Rosconi et al., 2015).

Since iron uptake systems are important for the endophytic lifestyle of *H. seropedicae* as for many bacterial endophytes, the aim of the present work was to have a more global picture of iron acquisition systems used by this model endophyte. For this purpose, we applied RNA-seq, RT-qPCR, proteomics approaches, and gene reporter constructs to identify genes differentially expressed under iron-limited conditions. Using these approaches, we identified genes related to the serobactins mediated iron uptake system, indicating the accuracy of our strategy, but we also were able to identify genes encoding several TonB-dependent receptors and inner membrane proteins hypothetically involved in iron transport. A triple mutant in the TonB-dependent receptors identified by mass spectrometry was constructed. Plant colonization was not altered in this mutant, suggesting that more transporters, not identified in our *in vitro* approach, could be active inside plants. Serobactin production by a mutant of Hsero_2337, a TonB-dependent receptor, showed that this protein acts in the transcriptional regulation of the serobactin biosynthetic genes probably by acting as a TonB-dependent transducer. The results of this work increased our understanding of the responses to iron deficiency in *H. seropedicae*, the only bacterial endophyte in which these systems have been described.

## Materials and Methods

### Bacterial Strain and Growth Conditions

Wild type, mutant strains and plasmids used in this work are listed in Supplementary Table S1. *H. seropedicae* strains were grown in minimal medium NFbHP-malate (Klassen et al., 1997) or TY (5 g/l tryptone, 3 g/l yeast extract, 0.05 g/l CaCl_2_; Beringer, 1974) at 30°C. *Escherichia coli* was grown in LB medium (Bertani, 1951) at 37°C.

Bioassays experiments were carried out as previously described (Amarelle et al., 2008) in TY medium with 400 μM of the metal-chelator ethylenediamine-di-*o*-hydroxyphenylacetic acid (EDDHA), at 30°C for 24 h. In each well, 10 μl of a different iron source were added. The iron sources tested were 37 mM FeCl_3_ as positive control, and 0.5 mM ferrichrome, 2 mM ferric dicitrate, and 300 nM ferric serobactin. Serobactin siderophores were purified from a culture of *H. seropedicae* Z67 wild-type strain as previously described (Rosconi et al., 2013).

Siderophore production was evaluated as the formation of an orange halo using the chromo azurol sulfonate (CAS) agar plate method (Schwyn and Neilands, 1987). Briefly, 10 μl of late exponential phase culture containing 1 × 10^6^ colony forming units (CFU) were spotted on CAS solid medium and incubated for 24 or 48 h at 30°C.

### Total RNA Extractions

Transcriptomic approaches were made in NFbHP-malate medium. *H. seropedicae* Z67 primary culture without iron addition was grown until it reached an optical density (OD) of 1.5. Then, secondary cultures with different iron availability conditions were inoculated with it, at an initial OD = 0.5. At 30, 60, 120, and 180 min cell samples of 10 ml were pelleted and stored at 4°C in RNAlater^®^ until RNA extraction. Working conditions were as follows: (a) 37 μM FeCl_3_, (b) without iron added, and (c) 75 μM 2′2-dipyridyl (DP).

RNA extractions were carried on using the Trizol method (Chomczynski, 1993). In brief, cells were resuspended in lysis buffer (50 mM Tris–HCl pH 8, 10 mM EDTA pH 8, 0.5% SDS). After the addition of Trizol reagent and chloroform, samples were centrifuged, and the aqueous phase was transferred to a new tube. These fractions were precipitated with isopropanol, incubated at room temperature, and centrifuged at 13,000 *g* for 10 min. RNA pellet was washed with 70% ethanol, dried, resuspended in nuclease-free water, and stored at -70°C. RNA extractions were treated with DNAse I to eliminate any contaminating genomic DNA. RNA concentration was estimated using a spectrometer (NanoDrop). The integrity of purified RNA was evaluated by visualization on an agarose gel and in an Agilent 2100 Bioanalyzer RNA chip.

### RNA-Seq: Sample Preparation and Data Analysis

RNA-seq was done only with the RNA extractions at 60 min. For rRNA depletion, we used the MICROB Express Kit^TM^ (Ambion), following manufacturer’s specifications. Starting from 7 μg of RNA of each sample, cDNA libraries were constructed using ION Total RNA-seq kit v2 for whole transcriptome libraries (Life Technologies), following manufacturer’s protocols. Correct size of cDNA libraries was analyzed in an Agilent 2100 Bioanalyzer chip. Sequencing was performed in an ION Proton semiconductor sequencer (Life Technologies) generating single-ended 100-basepair reads. Two runs were made with two biological replicates. The filtered reads, based on quality and length, were mapped to the reference genome of *H. seropedicae* SmR1 (NC_014323.1) using CLC Genomic Workbench 6.5.1. Expression values were normalized and reported in RPKM (reads per kilobase per million mapped reads). We considered two genes as differentially expressed when the absolute value of their fold-change was 2 or higher. Statistical analysis was performed using GraphPad Prism version 7.0 days for Mac OS X, GraphPad Software, La Jolla CA, United States^1^. We applied a multiple *t*-test with false discovery rate (FDR) approach correction (Benjamini, Krieger and Yekutieli two-stage step-up method) and without correcting for multiple comparisons (α = 0.005). We didn’t consider those genes with RPKM = 0 in one of the replicates and RPKM > 5 in the other replicate. In the case when in one of the compared conditions the RPKM was 0 in both replicates, we consider only those genes with a mean RPKM > 10 in the other condition to be considered as differentially expressed. A minimum read coverage of threefold was considered for a gene to be expressed (number of mapped reads multiplied by read length and divided by the gene length).

### RT-qPCR

cDNA was generated from 2 μg of each purified RNA, from the three condition extractions at all times (30, 60, 120, and 180 min), using the High Capacity RNA-to-cDNA kit^TM^ (Life Technologies). By manual search in the genome, we found and selected three genes thought to be involved in iron uptake mechanisms. To detect and quantify them, we designed specific primers using Primer Express 3.0 software (Applied Biosystems), which are listed on Supplementary Table S2. Primers efficiency was tested with qPCR using a pool of all cDNA samples as template in different serial dilutions (1:10, 1:5, and 1:2). 16S rDNA gene was used as normalization gene. As negative controls we used RNA samples, not treated with reverse transcriptase enzyme. Quantitative PCR was performed in StepOnePlus^TM^ Real-Time PCR System (Applied Biosystems). Amplification reactions were carried out in a final volume of 5 μl containing 2.5 μl of 2× Power SYBR^®^ Green PCR Master Mix (Life Technologies), 1 μM forward primer, and 1 μM reverse primer for each gene, and 1 μl of cDNA 1:5 dilution. The amplification protocol consisted on an initial incubation at 95°C for 10 min, followed by 40 cycles of 95°C for 15 s, and 60°C for 1 min. PCR runs were analyzed using automatic software settings. We made relative expression quantification (RQ) of the genes using the 2^-ΔΔCt^ method (Livak and Schmittgen, 2001), with +Fe 30 min as calibrating condition.

### Total Membrane Proteins Extraction, SDS-PAGE, and Identification by MALDI-TOF

Proteomic assays were made in NFbHP-malate medium. Primary culture (5 μl) without iron added and with an OD of approximately 1 were used to inoculate flasks containing 50-ml of media supplemented with 37 μM FeCl_3_ or 50 μM DP. Secondary cultures were incubated for 12 h until they reached early stationary phase (OD = 1).

Total membrane protein enriched fractions were obtained by centrifugation at high acceleration of cellular lysates as previously described (Rosconi et al., 2015). Aliquots of each sample were run on a 12% acrylamide SDS-PAGE gel, at 30 mA for approximately 2 h. Bands differentially expressed were cut and identified by peptide mapping by MALDI-TOF/TOF, using the service of the UByPA Unit at Institut Pasteur of Montevideo, Uruguay. The results were analyzed using the Mascot search engine^2^.

### Construction of Plasmid and Mutants

Strains and plasmids used in this study are listed in Supplementary Table S1, and primers in Supplementary Table S2. A mutant strain unable to produce FecA protein was constructed by interrupting the *fecA* gene with the *lacZaacC1* cassette obtained from plasmid pAB2002 (Becker et al., 1995). First, the *fecA* gene was amplified by PCR using FecA1 and FecA2 primers and cloned into the EcoRV site of pBluescript SK+^®^ (pBSK, Stratagene), generating plasmid pB*fecA*. Then, pB*fecA* was digested with EcoRV and then ligated with the *lacZaacC1* cassette, generating the plasmid with the interrupted gene (pB*fecA::lacZaacC1*), which then was sub-cloned in the BglII restriction site of suicide plasmid pWS233 (Selbitschka et al., 1993) (pWS*fecA*::*lacZaacC1*).

In-frame deletion mutation in Hsero_3255 (*fiu*) and Hsero_2720 (*ftr*) genes were constructed by crossover PCR (Horton et al., 1989), followed by two events of homologous recombination. Two rounds of amplifications were done with *H. seropedicae* genomic DNA using Pfu polymerase (Fermentas). The first round was done with primer pairs FiuP1-P2 and FiuP3-P4 for *fiu*, or FtrP1-P2 and FtrP3-P4 for *ftr*. The second round of amplification was performed with P1-P4 pairs, obtaining unique fragments of c.a. 500 bp flanking region of the *fiu* or *ftr* genes in a single product. These products were cloned in the EcoRV restriction gen site of the cloning vector pBluescript SK+^®^ (pBΔ*gene*). The Δ*gene* region was excised from this construction by a digestion with EcoRI/XbaI or just EcoRI and the fragment was sub-cloned in the suicide plasmid pWS233 (pWSΔ*gene*).

The entire gene Hsero_2337 (*cirA*) was amplified using primers CirA1 and CirA2. PCR product was double digested with XbaI/XhoI enzymes and cloned in the same sites of pBluescript SK+^®^ (pB*cirA*). Using this constructed plasmid as template, an inverted PCR was performed with primers CirA3 and CirA4 using Pfu polymerase (Fermentas). The obtained product was re-circularized (pBΔ*cirA*), digested with PstI, and cloned in the same site of plasmid pAB2001 (Becker et al., 1995) generating plasmid pA1Δ*cirA*. This last construct was digested with BamHI and cloned in the BglII site of plasmid pWS233 (pWSΔ*cirA*). Complementation plasmid pC*cirA* was constructed by sub-cloning a PstI digestion fragment from pB*cirA* containing the gene in the same site of plasmid pCPP30 (Huang et al., 1992).

To obtain a Hsero_2339 mutant construct, *pfrI* gene was amplified with Pfu polymerase using primers PfrI1 and PfrI2 and cloned in the EcoRV site of pBluescript SK+^®^ (pB*pfrI*). A *lacZaacC1* cassette was obtained after digestion of pAB2001 with SmaI and cloned in an EcoRV site present in the middle of gene Hsero_2339 (pB*pfrI::lacZaacC1*). A BamHI-HindIII fragment from this construct containing the interrupted gene was cloned in the same site of pAB2002 (pA2*pfrI::lacZaacC1*), and finally this plasmid was digested with EcoRI and the fragment containing the interrupted gene was cloned in the same site of the pWS233 (pW*pfrI::lacZaacC1*). Complementation plasmid pC*pfrI* was constructed by sub-cloning a HindIII-EcoRI digestion fragment from pB*pfrI* containing the gene in the plasmid pCPP30 digested with the same enzymes.

Plasmid p237*mbth*pr, which contains the promoter region of gene Hsero_2339 (*mbtH*) controlling the expression of the reporter gene cyan fluorescent protein (CFP), was obtained as follow: intergenic region between *pfrI* and *mbtH* was amplified with Pfu polymerase using primers MbtHpr1 and MbtHpr2 and cloned in the EcoRV site of pBluescript SK+^®^ (pB*mbtH*pr). This plasmid was digested with EcoRI-XbaI and cloned in the same sites of plasmid pSEVA237-C (Martinez-Garcia et al., 2015).

The constructs were introduced in *H. seropedicae* Z67 strain by triparental mating as previously described (Rosconi et al., 2015), using the *E. coli* TOP10 strain containing the constructed plasmids and the *E. coli* TOP10 strain containing a pRK2013 as helper plasmid (Ditta et al., 1980). Mutants constructed using pWS*gene* plasmids were obtained by two events of homologous recombination, selecting first the simple recombination events in TY 5 μg/μl nalidixic acid and 5 μg/μl tetracycline and double recombination events in NFbHP-malate medium with 8% saccharide (Rosconi et al., 2015). Deletions and insertions were confirmed by colony PCR, using the correct designed primers for each gene and in the case of *fecA* mutant also by Southern blot analysis of SacI-digested genomic DNA using a biotinylated probe of wild-type *fecA* gene (Sambrook et al., 1989).

Double and triple mutants generated in this work were constructed by successive matings in the appropriate parental strains with the appropriate plasmids carried in *E. coli* TOP10 donors and a *E. coli* TOP10 pRK2013 as a helper strain.

Restriction enzymes were purchased from Promega, and ligation reactions were performed using T4 ligase (Invitrogen). Plasmid preparation by alkaline lysis, preparation of competent cells, and transformation by heat shock were performed by standard protocols (Sambrook et al., 1989). DNA electrophoresis was carried out on 0.8% (w/v) agarose gel and visualized by staining with SYBR Safe^®^ (Thermo) and exposure to UV light. DNA purification from agarose gels was made using the QIAquick Gel Extraction Kit^®^ (QIAGEN).

### Gene Expression Assays

Transcriptional activity of *fecA* gene was evaluated using β-galactosidase activity using the *lacZ* cassette present in Z67-*fecA* mutant. Assays were performed essentially as previously described (Miller, 1972), with few modifications (Rosconi et al., 2006). Strains were grown in NFbHP-malate medium with (a) 37 μM FeCl_3_, (b) 50 μM EDDHA, (c) 50 μM EDDHA 0.1 μM ferric dicitrate, (d) 50 μM EDDHA 1 μM ferric dicitrate, (e) 50 μM EDDHA 10 μM ferric dicitrate, and (f) 50 μM EDDHA 100 μM ferric dicitrate.

Transcriptional activity of *pfrI* gene was also evaluated using β-galactosidase activity using the *lacZ* cassette present in Z67-*pfrI* mutant but using a 96-well plate protocol previously described (Rosconi et al., 2015). Different conditions evaluated were NFbHP-malate medium with 37 μM FeCl_3_ or 50 μM DP.

Transcriptional regulation of the Hsero_2339 gene was determined using the CFP as a reporter gene contained in plasmid p237*mbtH*pr. Strains were grown in Cell Star^®^, Greiner Bio One 96 well plates, and incubated at 30°C in a rotary shaker until early stationary phase. The plate was loaded in a Varioskan Flash^®^ (Thermo), OD was determined by measuring the absorbance at 620 nm, and CFP concentration was measured by exciting the plate wells at 435 nm and recording the emission at 485 nm. Expression arbitrary units were normalized and presented as fluorescence/OD ratio. Different conditions evaluated were NFbHP-malate medium with 37 μM FeCl_3_ or 50 μM DP.

Results are the average of three independent experiments with at least four replicates in each experiment.

### *In Vitro* Biofilm Formation

Crystal violet assays were performed as described before (Villegas et al., 2013). Pre-cultures in TY medium were incubated in U-bottom Cell Star^®^ Greiner Bio One 96-well plates at 37°C overnight. Second cultures were inoculated in TY medium with different iron availability conditions, each well grown in triplicate, in flat-bottom Cell Star^®^ Greiner Bio One 96-well plates. The different iron availability conditions were obtained with the addition of 37 μM FeCl_3_ or 200 μM DP. Plates were incubated at 37°C for 48 h. After crystal violet stain, wash, and solubilization of CV with 95% ethanol, the absorbance was measured in a spectrophotometer Varioskan^TM^ Flash Multimode Reader (Thermo Scientific). Results are expressed as the average of three independent experiments, each with three wells per condition tested. Statistical differences were evaluated by Duncan’s test with a *p*-value of 0.05. The classification of the strains was done considering the scale based on Villegas et al. (2013) for this OD 590 nm: less than 0.21 is not considered a biofilm producer; from 0.21 to 0.42 weak producers; from 0.42 to 0.84 moderate biofilm producers; and more than 0.84 strong producers.

### Plant Colonization Assays

Colonization assays of rice plants grown in gnotobiotic conditions and bacterial recovery from plant-aerial parts were essentially as describe before (Alberton et al., 2013). Seeds from *Oryza sativa* cv. Tacuarí (generously provided by Sebastián Martínez, from INIA, Treinta y Tres, Uruguay) were used. For plant inoculation, axenic seedlings were incubated for 30 min with a bacterial suspension containing 1 × 10^6^ CFU/ml of *H. seropedicae* Z67 wild type strain or the derived mutant strains. Inoculated seedlings were transferred to 25 ml of carbon-free Hoagland nutrient solution (Hoagland and Arnon, 1950) pH 6.5-7.0, and incubated for 8 days at 26°C and a 16-h light photoperiod. After that, plant-aerial parts were superficially sterilized as explained by Alberton et al. (2013), macerated in 1 ml of saline solution, and serial dilutions were plated onto solid NFbHP-malate for CFU counts. Results are expressed as the average of three independent experiments, each with five tubes per condition tested. Statistical differences were evaluated with a *p*-value of 0.05.

### Confocal Microscopy Observations

Sterilized rice seedlings inoculated with wild-type or Z67-*sbtI*/*sbtR* mutant strains were grown as described above. At eight days post-inoculation (PI), plants were harvested and cuts performed both on roots and aerial parts using scalpel (thick cuts) or cryostat (Leica CM1800) (thin cuts). Cuts were stained by incubating them for 15 min in a wet chamber in the presence of 12 μM Syto9 Stain (Life Technologies), a DNA intercalating agent. Cuts were mounted in slides and acquisition of 3D images was performed by a confocal laser scanning microscopy (CLSM) Olympus BX-61 direct FV300, 100× immersion objective, N.A. 1.35 and 488 nm/520 nm excitation/emission wavelength. Images obtained in *xy*-axis of 1024 × 1024 pixels and Z-stacks acquired with a step size of 0.3 μm were reconstructed using Volocity 3D Image Analysis Software (Perkin Elmer). Experiments were carried out with three tubes as biological replicates, each containing three plants, and four cuts per plant were observed.

### *In Silico* Analysis and Visualization Tools

**FIGURE 1:**
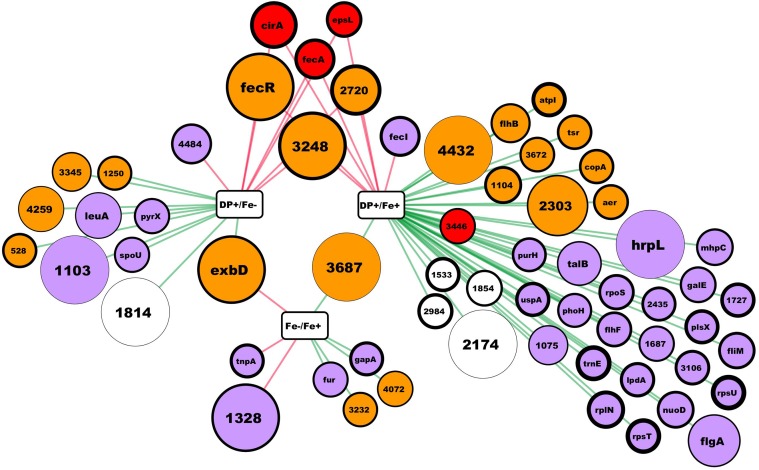
RNA-seq results summary. In this network, rectangular nodes correspond to the experimental conditions compared, circular nodes correspond to genes (named by gene symbol or by the # in the Hsero_# locus tag) differentially expressed according to our criteria. Red edges connect a conditions comparison with the up-regulated genes, while green edges connect the down-regulated ones. Nodes colors represent putative protein cellular localization: red outer membrane, orange inner membrane, light violet cytoplasmic, blank not enough information. Nodes diameter correlate with the absolute fold change from the comparison connected by an edge. In the case of nodes connected to two comparisons, fold change is the one from the comparison with the higher difference in iron availability between conditions. Nodes border thickness correspond to the Log_2_ of the mean RPKM of the condition in the comparisons nodes (rectangles) with the lower iron availability.

Differentially expressed genes features were obtained at the Integrated Microbial Genomics platform at http://img.jgi.doe.gov/ (Markowitz et al., 2010). **Figure 1** was created using Cytoscape 3.6.1 (Shannon et al., 2003).

## Results

### Iron Deficiency Differentially Expressed Genes Identified at the RNA Level

As described in many bacteria, the main response to iron deficiency starts at the transcriptional level by the de-repression and activation of different genes usually through the action of different master regulators (O’Brian and Fabiano, 2010). Through RNAseq, we aimed to obtain a global knowledge of the genes tendency to be repressed or over-expressed under iron limiting conditions in *H. seropedicae*. Using the annotated *H. seropedicae* SmR1 as the reference genome, we mapped the reads, assembled the transcripts, and analyzed the expression differences (Supplementary Table S3). **Figure 1** depicts the results obtained in the RNAseq where we consider as differentially expressed those genes with an absolute fold change higher than 2 and an uncorrected *p*-value lower than 0.005. When comparing expression of genes between NFbHP-malate with or without iron added only eight genes matched our criteria (five down and three up-regulated in the condition without iron). These few observed differences suggest that iron traces in the growth medium may be sufficient for *H. seropedicae* to keep high affinity iron-uptake systems off. But, when we compared these two conditions with the one with the chelating agent DP (**Figure 1** and Supplementary Table S3), we found several genes with an expression fold-change higher than 2. When comparing the condition against the media without added iron, the number of differentially expressed genes is 17 (10 down and 7 up-regulated in the condition with DP). In the case of the medium with DP against medium with added iron, the number of differentially expressed genes increases to 45 (38 down and 7 up-regulated). Six genes showed to be up-regulated when comparing the DP containing medium against the other two. These genes included two coding for outer membrane TonB-dependent receptors: Hsero_1277 (*fecA*), homolog to ferric dicitrate transporters and Hsero_2337 (*cirA*), which codes for a putative TonB-dependent receptor encoded in the serobactin biosynthetic cassette with unknown function in the iron-serobactin acquisition system (Rosconi et al., 2015). The other three of these six genes are probably inner membrane proteins: Hsero_2720, a gene encoding a putative Fe^2+^/Pb^2+^ high affinity permease homolog to Ftr; Hsero_3248, described as a gene coding for an iron-regulated membrane protein; and gene Hsero_0084 which codes an anti-sigma protein annotated as FecR. Finally, Hsero_1987 (*epsL*), the first gene of a cluster involved in EPS biosynthesis was also over expressed in the presence of DP. This gene contains a putative beta-barrel porin 2 domain (pfam 10082) and a signal peptide (Supplementary Table S3), which suggests it is an outer membrane protein. Considering the serobactins biosynthetic genomic locus (Hsero_2337 to Hsero_2349), only *cirA* appeared as differentially expressed according our criteria. However, we can’t state that the rest of the genes are not being expressed, since the RPKMs for all of those genes in the DP medium are much higher than the RPKMs in the other two conditions (Supplementary Table S3), but very different between the two replicates.

**FIGURE 2:**
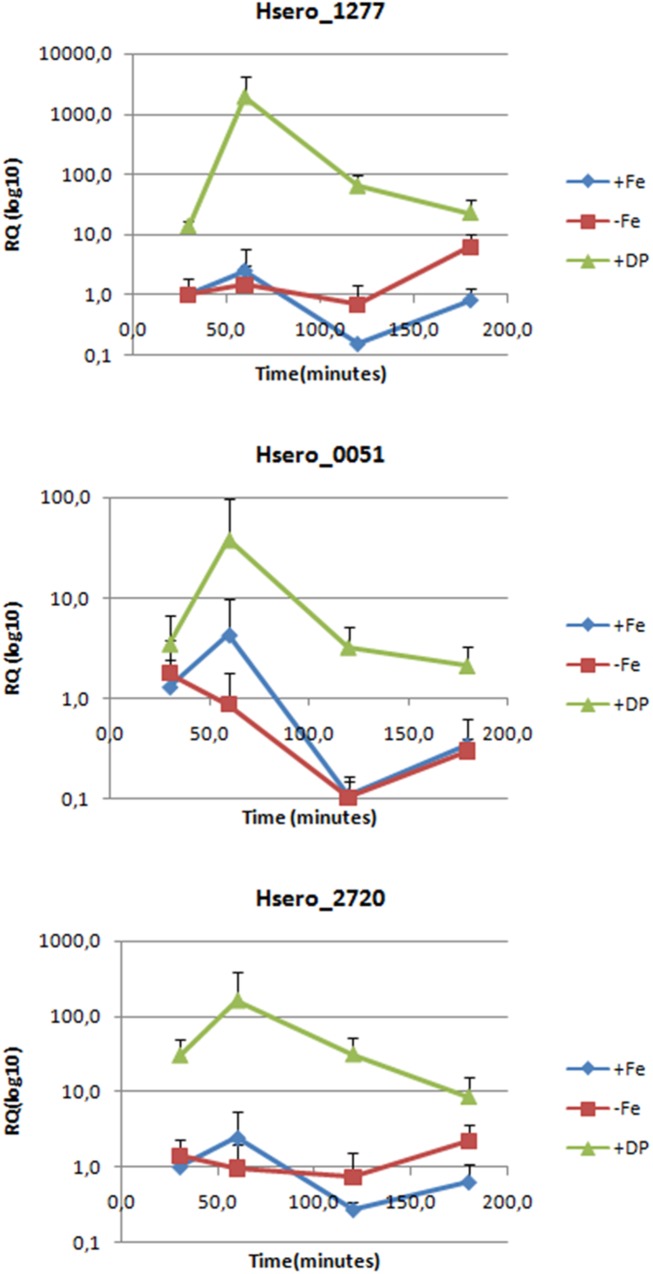
Expression levels of three genes probably involved in iron metabolism measured by RT-PCR. Relative quantification is expressed as base-10 logarithm (RQlog10) for each gene at each assayed time (30, 60, 120, and 180 min), using the 2^-ΔΔCt^ quantifying method; the 16S rDNA as the normalization gene and +Fe 30 min as the calibrator condition. Standard deviation of the three biological replicates is indicated in each bar.

To confirm RNAseq results, RT-qPCR genes expression analyzes were performed for Hsero_2720 coding a high affinity Fe^2+^/Pb^2+^permease (*ftr*) and Hsero_1277 coding for the ferric dicitrate transporter (*fecA*). Hsero_0051 (*feoB*), coding for a putative ferrous iron transport transmembrane protein was included in this analysis, because it was identified by *in silico* search in the reference genome of *H. seropedicae* SmR1. The relative quantification by RT-PCR of gene expression under different iron conditions and at different times is summarized in **Figure 2**. Results confirmed that *fecA* and *ftr* were over-expressed in iron-limited conditions, but they also showed that *feoB* gene was up-regulated (in RNAseq studies it had a fold-change of 5.87, but its *p*-value was of 0.2). There is also a marked peak in the expression of these three genes at 60 min of incubation, which decayed at 180 min.

Considering the results obtained in the RNA studies, we chose genes Hsero_1277 (*fecA*), Hsero_2337 (*cirA*), and Hsero_2720 (*ftr*) to construct mutants and phenotypically characterize them, as described in following sections.

### Iron Deficiency Differentially Expressed Membrane Proteins

Since most of the identified genes in our transcriptomic approach are membrane proteins (**Figure 1**), we performed a proteomic approach to identify membrane proteins induced under iron starvation conditions at the initial stationary phase.

**FIGURE 3:**
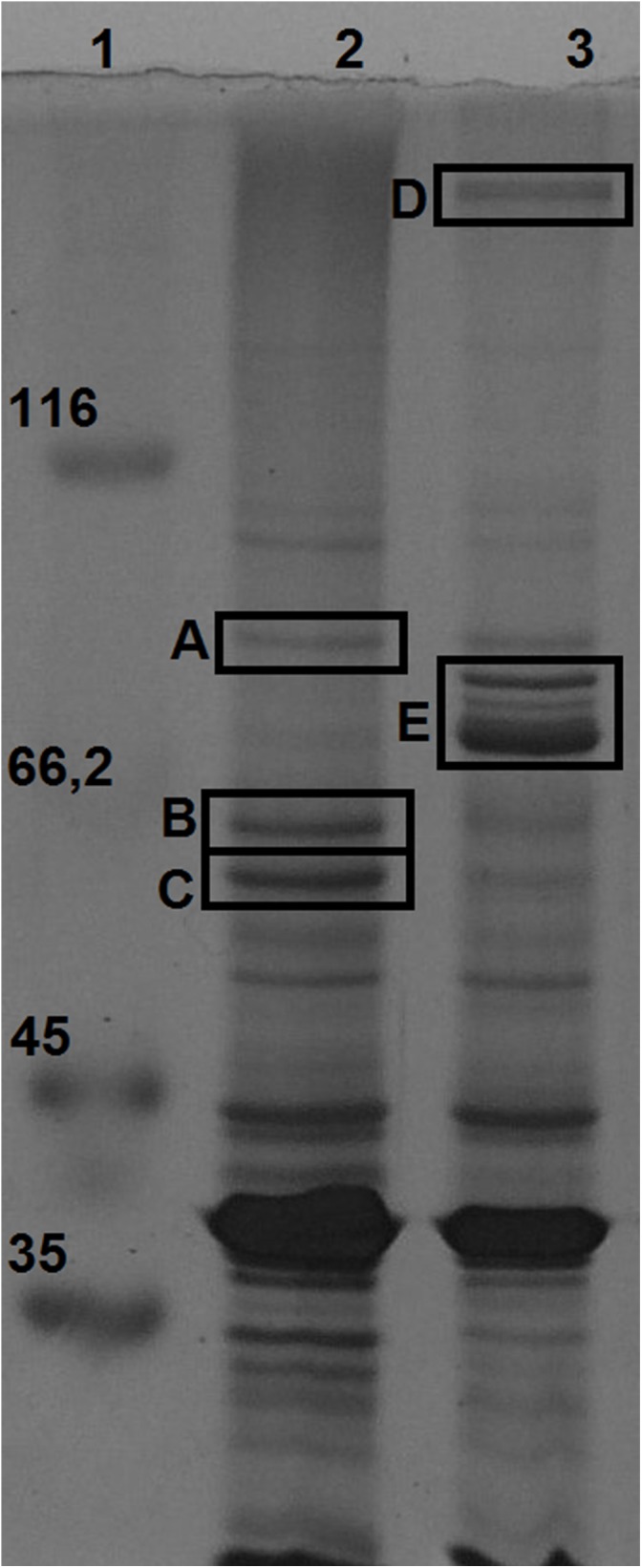
Membrane proteins regulated by iron availability at late exponential growth phase. SDS-PAGE 12% acrylamide gel of total membrane protein enriched fractions of bacteria grown in NFbHP-malate media with different iron conditions: 37 μM FeCl_3_ (lane 2); 50 μM 2′2-dipyridyl (lane 3). Molecular weight marker in KDa (lane 1). Letters from **(A–E)** are bands differentially expressed, identified by MALDI-TOF as: Hsero_4295 **(A)**; Hsero_2973, *sdhA* **(B)**; Hsero_0972 **(C)**; Hsero_2343, *sbtI* **(D)**; Hsero_3255 (*fiu*), Hsero_2345 (*sbtR*) and Hsero_1277 (*fecA*) **(E)**.

Membrane proteins enriched fractions of *H. seropedicae* Z67 cultures grown with high and low iron availability (37 μM FeCl_3_ and 50 μM DP, respectively) were analyzed by SDS-PAGE. Seven bands with differential expression intensities were selected for identification by peptide mapping in a MALDI-TOF mass spectrometer (**Figure 3**). Three bands that migrate very close in the low iron availability environment were analyzed as a single sample in the MALDI-TOF, and are collectively referred to as band E. As a control we included one band with similar intensity in both conditions, which was named band A and was shown to be the product of the gene Hsero_4295, an outer membrane porin identified recently as a candidate essential gene of *H. seropedicae* (Rosconi et al., 2016). Band B, highly expressed in iron sufficient condition, was identified as the product of Hsero_2973, a homolog of *sdhA*, encoding the succinate dehydrogenase flavoprotein subunit that contains a Fe-S cluster (McNeil et al., 2014). Band C corresponded to another protein with induced expression under iron sufficient conditions; it was identified as the one coded by the gene Hsero_0972, a sugar ABC transporter periplasmic protein. On the other hand, four proteins were identified as over-expressed in iron limiting condition: the NRPS SbtI (Hsero_2343) (band D); the serobactin receptor SbtR (Hsero_2345), a TonB-dependent outer membrane receptor for iron monomeric catechols annotated as *fiu* (Hsero_3255), and the outer membrane ferric dicitrate transport protein FecA (Hsero_1277) (the latest three genes in band E). *fecA* was previously identified as iron regulated by RNAseq and qRT-PCR. The fact that we identified the NRPS SbtI as a membrane associated protein, suggests that biosynthesis of serobactins is membrane located, as occurs with *Pseudomonas aeruginosa* siderophores pioverdins and their described siderosomes (Gasser et al., 2015). Under the assayed growth conditions, no inner membrane transporters were identified by this approach suggesting that iron may be transported to the cytoplasm by non-differentially expressed systems. In the light of these results we decided to include mutants of the gene Hsero_3255 (*fiu*) to the functional studies.

### Mutants’ Phenotypic Studies

In order to clarify the importance of the different iron-uptake systems in the growth behavior of *H. seropedicae*, the wild type strain and some selected mutants (Supplementary Table S1) were characterized phenotypically. Phenotypic analyses included bioassays and biofilm formation. In the case of *fecA* mutant, we exploited the *lacZ* insertion (*fecA::lacZaacC1*) to study its regulation by analyzing β-galactosidase activity (Supplementary Figure S1). Citrate is a molecule with a moderate affinity for iron used by some bacteria as an iron source and by vascular plants to transport iron along the xylem (Braun et al., 2006; Rellan-Alvarez et al., 2010). As *fecA* is annotated as a ferric dicitrate transporter, we tested its expression at different ferric dicitrate concentrations. We noticed that at low concentration of ferric dicitrate (0.1–10 μM), *fecA* had high expression levels, while as we increased the ferric dicitrate concentrations its expression diminished. On the other hand, bioassays experiments showed no growth differences between wild-type strain and *fecA* or *fiu* mutants, all showing growth halos around ferric dicitrate (Supplementary Figure S2). In these bioassays experiments we also tested ferrichrome and ferric serobactin as iron nutritional sources. Ferrichrome is an abundant molecule in soil produced by various fungi (Olofsson Madelen and Bylund, 2015). All of the mutants grew in the presence of ferrichrome. As expected, only mutant strains lacking the *sbtR* gene, were unable to grow at expenses of serobactins. The strain that showed the most compromised *in vitro* growth under low iron availability liquid medium was the mutant in the three TonB-dependent receptors *fecA/sbtR/fiu*; however, this growth defect was not much lower than the one presented by the mutant Z67-*sbtR* (data not shown).

We previously demonstrated that iron acquisition systems mediated by serobactins in *H. seropedicae* confer competition fitness inside rice plants (Rosconi et al., 2015). But these results also suggested that *H. seropedicae* is using different iron uptake mechanisms when passing through an endophytic lifestyle since the serobactin minus mutant is still able to colonize and survive inside rice plants. So we tested the ability of different mutants to effectively colonize rice plants and, by using confocal microscopy, we looked for bacteria inside the plant. After 8 days of individually inoculation, *H. seropedicae* wild-type strain was able to colonize the aerial parts of rice in the order of 10^7^ CFU/g of fresh weight. At the same time, mutant strains were recovered in the order of 10^5^–10^7^ CFU/g of fresh weight from the interior plant, although values were not statistically different due to variability of assays (Supplementary Figure S3). Confocal microscopy analyses clearly shown that both wild type and *sbtI/sbtR* strains form similar biofilm-like bacterial aggregates in plant surfaces (Supplementary Figure S4). Iron availability was reported to differentially affect biofilm formation in different bacteria (Wu and Outten, 2009). So we aimed to test the ability of biofilm formation by *H. seropedicae* Z67 and derived mutants described in this work. Although the *in vitro* biofilm formation assay presented high variation, results showed that *H. seropedicae* is a moderate biofilm producer (0.5 a.u., *SD* = 0.3). However, this ability is not affected by iron availability nor genetic context.

### CirA and PfrI Role in the Production of Serobactins

**FIGURE 4:**
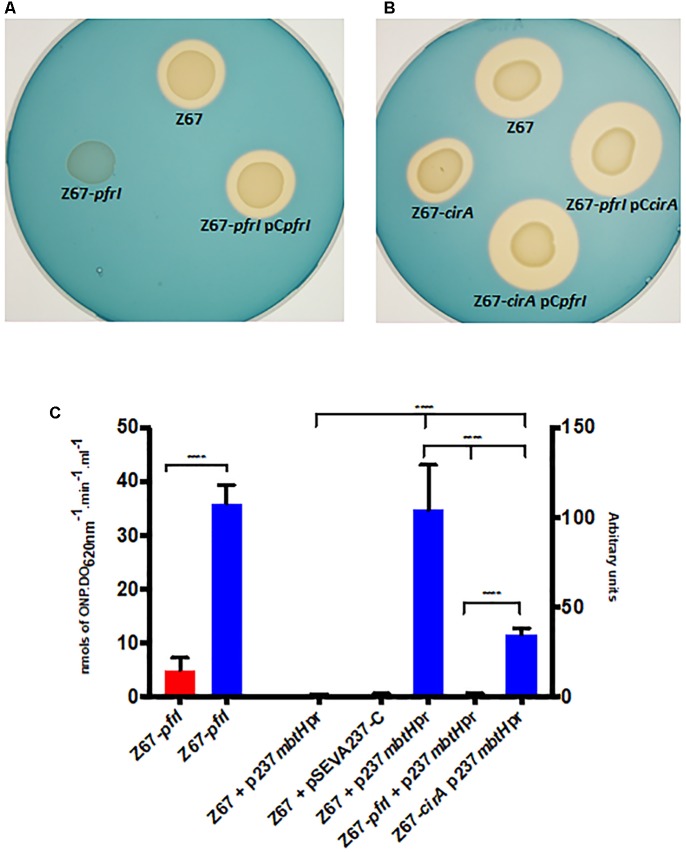
Phenotypic characterization of mutant strains Z67-*pfrI* and Z67-*cirA*. Serobactins production/internalization of *H. seropedicae* Z67 wild-type strain, Z67-*pfrI* **(A)** and Z67-*cirA* **(B)** mutant strains on solid CAS medium with or without complementing plasmids as indicated. Ten microliters of a late exponential phase culture containing 1 × 10^6^ CFU were spotted onto the CAS solid medium and incubated for 24 h **(A)** or 48 h **(B)** at 30°C. **(C)** Left: β-galactosidase activity of the Z67-*pfrI* mutant strain growing in NfbHP-malate with 37 μM FeCl_3_ (red bar) or with 50 μM DP (blue bar). Right: CFP expression of different strains containing the plasmid p237*mbtH*pr or the empty vector pSEVA237-C. The expression of all strains in NfbHP-malate with 37 μM FeCl_3_ presented similar values close to 1 and similar to the wild type strain with the empty vector in the same condition. Only the wild type is represented (Z67 p237*mbtH*pr, third bar). Z67 pSEVA237-C bar correspond to the expression of this strain in NfbHP-malate with 50 μM DP. The remaining three bars correspond to the expression of CFP in the indicated strain in NfbHP-malate with 50 μM DP. Results are the average of at least three independent experiments with four or more technical replicates each. Bars represent the 95% CI of the mean. Statistical analysis was performed using ordinary One-way ANOVA without correction for multiple comparisons. ^∗∗∗∗^*p* < 0.0001.

Two TonB-dependent receptors for siderophores, coded by Hsero_2337 and Hsero_2345, appear in the serobactin biosynthesis genomic cassette. One of them, SbrT (Hsero_2345), we previously demonstrated that is responsible for serobactin internalization. The other, CirA (Hsero_2337) was identified in this work as differentially expressed in the RNAseq experiments. These ORFs have 63% identity between them. However, CirA has a STN domain (Pfam 07660), characteristic of TonB-dependent transducers, which is absent in SbrT. A probable ORF homolog to the iron starvation extra cytoplasmic sigma factor *pvdS* (*pfrI*) is located downstream the *cirA* gene. No homologs for a cognate anti-sigma factor is present in this region. Interestingly, CirA was not identified in the enriched membrane fractions of our proteomic approach. In order to shed light on CirA function we constructed knock out mutants in the *cirA* and *pfrI* genes as well as complementation plasmids (Supplementary Table S1). Then, we analyzed mutants’ phenotype in CAS medium (**Figures 4A,B**). In mutant strain Z67-*pfrI* serobactin production is completely abolished, while the mutant Z67-*cirA* CAS halo is less than half than the one of the wild type, suggesting that serobactin production is reduced. Both mutants are complemented *in trans* with plasmids containing the respective intact genes, but also, mutant Z67-*cirA* is complemented by the plasmid containing the intact copy of *pfrI* (pC*pfrI*). It was previously reported that mutants in TonB-dependent transducers can be complemented by increasing the copy numbers of the cognate sigma factor (Llamas et al., 2008).

Genes Hsero_2339 to Hsero_2343 code for serobactins biosynthetic genes. In the upstream region of gene Hsero_2339 (*mbtH*), we found a canonical IS box (TAAAT-N16-CGT), the recognition sequence for the extracytoplasmic sigma factors PvdS of *Pseudomonas aeruginosa* (Tiburzi et al., 2008). We cloned the promoter region of *mbtH* containing the IS sequence in front of a promoter-less CFP. We introduced this construct (pSEVA*mbtH*pr) in Z67 wild type and in the Z67-*pfrI* and *cirA* mutant strains (**Figure 4C**). In the wild type context, CFP expression is only activated under low iron conditions, in mutant Z67-*pfrI* context the fluorescent protein is not expressed at all, and in the mutant Z67-*cirA* context expression in low iron conditions is reduced by more than half of the wild type one. These results show that serobactin production is totally dependent on PfrI and that it needs CirA to reach a maximum expression. As expected, *pfrI* is also expressed under low iron conditions (**Figure 4C**).

## Discussion

The high number of iron acquisition systems found in endophytes genomes including *H. seropedicae*, plus the results we published before on this bacterium led us to investigate in more depth the iron acquisition strategies of this endophyte. This work used various approaches to indentify several genes activated by *H. seropedicae* in response to iron limitation. Our results from RNA differential expression experiments showed us that most of the genes over-expressed in low iron conditions encodes for membrane-associated proteins (**Figure 1** and Supplementary Table S3). Hence, we proceeded to perform a proteomic approach where we identified membrane proteins regulated by iron availability. Differential expression of some of the genes identified by RNA-seq and some non-identified genes were validated and confirmed to be iron-regulated by RT-PCR or by using reporter genes. As expected, in our *in vitro* assays in which *H. seropedicae* grows isolated, serobactin-mediated acquisition systems appear to be the most successful strategy to overcome iron limitation. In our RT-PCR experiments (**Figure 2**), we showed that the initial response changed after 180 min. This could be due to the fact that iron is successfully taken by serobactins and the iron starvation inside cells becomes less stringent.

However, other two TonB-dependent receptors (FecA and Fiu) not related to iron-serobactin acquisition (Supplementary Figure S1) are also up-regulated in low iron conditions, and their expression is maintained even in early stationary phase, when serobactin acquisition systems are fully established. Usually, TonB-dependent receptors have two levels of regulation: first a master regulator, like Fur, Irr, RirA, de-represses certain signal transduction systems or transcriptional regulators that sense the presence of specific iron nutritional sources in the environment (O’Brian and Fabiano, 2010). If one of these specific sources is present, the signal transduction systems or the transcriptional regulator activate the expression of transport genes as TonB-dependent receptors. If this were the case for FecA and Fiu, the expression of some molecules present on the Nfb-malate medium or produced by *H. seropedicae* would be induced. But, it could be possible that both FecA and Fiu have only one level of regulation: de-repression when available iron is low. If this were the case, one plausible hypothesis would be that iron complexes transported by both proteins are readily available in the natural environment of *H. seropedicae*. Ferric citrate complexes are abundant in plant xylem and other plant tissues, and ferrichrome is abundant in soil. Although we showed that *H. seropedicae* can use both molecules as iron nutritional sources, FecA and Fiu are not involved or are not the only proteins involved in ferric citrate or ferrichrome transport. In *Mycobacterium smegmatis* for instance, ferric citrate is acquired through porins by diffusion (Jones and Niederweis, 2010), so maybe the same passive mechanisms can be acting on *H. seropedicae.* In the case of ferrichrome, many other TonB-receptor homologs to ferrichrome transporters are present in the *H. seropedicae* genome, and most of them are genetically linked to signal transduction systems or transcriptional regulators.

Results obtained from plant assays showed that the mutants we tested in this work successfully colonize the host plant. It is well known that flooded rice plants can form iron crusts on its roots (Huang et al., 2015). So, when growing at the root surface, *H. seropedicae* could be taking and storing iron and can be using this stored iron during its endophytic stage. This could explain why we couldn’t see phenotypes for the mutants in our experiments. However, we cannot rule out that iron uptake mechanisms mediated by Fiu and FecA may confer a competitive advantage for colonization of the plant niche, as occurs for the ones mediated by serobactins.

RNAseq experiments results showed that the TonB-dependent receptor CirA, coded by Hsero_2337, is expressed under low iron availability. However, in a previous work and in this one, this protein was not identified in fractions enriched in membrane proteins. We can hypothesize that CirA is not expressed at the early stationary phase when we isolated the membrane proteins, or CirA is expressed at a very low level, not detectable by silver staining on a SDS-PAGE gel. In any case, RNAseq results led us to investigate the role of CirA in iron-serobactin acquisition systems. From the results obtained by the phenotypic characterization of mutant strains Z67-*cirA* and Z67-*pfrI* phenotypic characterization (**Figure 4**), we propose a model where CirA acts as a TonB-dependent transducer that senses the feasibility of using iron-serobactins as nutritional sources. In a positive scenario, CirA inactivates a not identified anti-sigma factor that releases PfrI, which then activates at its maximum the production of serobactins. In all other bacterial species described, the TonB-dependent transducer also acts as a transporter, but in *H. seropedicae* these two functions are performed by two different proteins: CirA and SbtR.

## Conclusion

The future outcomes arising from this work could be focused in two directions: first, to identify and describe in detail the strategies used by *H. seropedicae* to acquire iron in the different stages of its host plant colonization. Competition assays between multiple mutants or *in vivo* gene expression analysis can shed light to achieve this goal. Second, it could be interesting to complete the description of the mechanisms of iron acquisition mediated by serobactins. As an example, we don’t know how iron is internalized to the cytoplasm after entering to the periplasm through SbtR. One plausible hypothesis would be that this role is fulfill by the concerted action of a ferric iron reductase (Hsero_2348) present in the serobactin biosynthetic genomic locus, in interaction with the ferrous iron permeases Ftr (Hsero_2720) and FeoB (Hsero_0051), both identified in this work as differentially expressed under low iron conditions.

## Author Contributions

MT contributed to the RNAseq, RT-qPCR, proteomics, mutagenesis, biofilm assays, plant assays, microscopic assays, experimental design, results analysis, and manuscript writing. PS contributed to the biofilm assays, microscopic assays, and manuscript correction. RP contributed to the mutagenesis, experimental design, and manuscript correction. EdS contributed to the experimental design, results analysis, and manuscript correction. EF contributed to the experimental design, results analysis, and manuscript correction. FR was the principal investigator and idea generator, and contributed to the experimental design, mutagenesis, results analysis, and manuscript writing.

## Conflict of Interest Statement

The authors declare that the research was conducted in the absence of any commercial or financial relationships that could be construed as a potential conflict of interest.

## Footnotes

1 www.graphpad.com

2 www.matrixscience.com
