# Supplementary material for: Herbaspirillum seropedicae Differentially Expressed Genes in Response to Iron Availability
Source: Front Microbiol. 2018 Jul 3;9:1430. doi: 10.3389/fmicb.2018.01430 (PMC6037834; doi:10.3389/fmicb.2018.01430)
Supplement: Supplementary file 1 [file Table_1.DOCX]

**Table S1**. Strains and plasmids used in this work.

| **Strains** | **Relevant characteristics** | **Reference** |
| --- | --- | --- |
| Z67 | *H. seropedicae* Z67 wild-type, *Nal^R^* | (Baldani et al., 1986) |
| Hs 4.3 | Z67 with *mTn5-gusA-o-pgfp* in Hsero_2343 | (Rosconi et al., 2013) |
| Z67-*sbtI* | Z67 with *lacZaacC1* cassette in Hsero_2343 | (Rosconi et al., 2015) |
| Z67-*sbtR* | Z67 with in frame deletion of Hsero_2345 | (Rosconi et al., 2015) |
| Z67- *sbtI*/*sbtR* | Z67 with *lacZaacC1* cassette in Hsero_2343 and in frame deletion of Hsero_2345 | (Rosconi et al., 2015) |
| Z67-*fecA* | Z67 with *lacZaacC1* cassette in Hsero_1277 | This work |
| Z67-*fiu* | Z67 with in frame deletion of Hsero_3255 | This work |
| Z67-*ftr* | Z67 with in frame deletion of Hsero_2720 | This work |
| Z76-*fecA*/*sbtR* | Z67 with *lacZaacC1* cassette in Hsero_1277 and in frame deletion of Hsero_2345 | This work |
| Z67- *sbtR*/*fiu* | Z67 with in frame deletion of Hsero_2345 and in Hsero_3255 | This work |
| Z67-*fecA*/*fiu* | Z67 with *lacZaacC1* cassette in Hsero_1277 and in frame deletion of Hsero_3255 | This work |
| Z67-*fecA*/*sbtR*/*fiu* | Z67 with *lacZaacC1* cassette in Hsero_1277 and in frame deletion of Hsero_2345 and in Hsero_3255 | This work |
| Z67-*sbtI*/*sbtR*/*fiu* | Z67 with *lacZaacC1* cassette in Hsero_2343 and in frame deletion of Hsero_2345 and in Hsero_3255 | This work |
| Z67-*sbtI*/*sbtR*/*fecA* | Z67 with *mTn5-gusA-o-pgfp* in Hsero_2343 (Hs 4.3), a  *lacZaacC1* cassette in Hsero_1277 and in frame deletion in Hsero_2345 | This work |
| Z67-*cirA* | Z67 with in frame deletion of Hsero_2337 | This work |
| Z67-*pfrI* | Z67 with *lacZaacC1* cassette in Hsero_2337 | This work |
| *E. coli* TOP10 | General cloning strain, *Str*^R^ | Invitrogen |
| **Plasmids** |  |  |
| pBluescript SK + (pBSK) | *Amp^R^, lacZ* | Stratagene |
| pB*fecA* | Hsero_1277 cloned in pBSK | This work |
| pB*fecA::lacZaacC1* | Hsero_1277 interrupted with *lacZaacC1* cloned in pBSK | This work |
| pB∆*fiu* | In frame deleted Hsero_3255 cloned in pBSK | This work |
| pB∆*ftr* | In frame deleted Hsero_2720 cloned in pBSK | This work |
| pB*cirA* | Hsero_2337 cloned in pBSK | This work |
| pB∆*cirA* | In frame deleted Hsero_2337 cloned in pBSK | This work |
| pB*pfrI* | Hsero_2338 cloned in pBSK | This work |
| pB*pfrI::lacZaacC1* | Hsero_2338 interrupted with *lacZaacC1* cloned in pBSK | This work |
| pB*mbtH*pr | Promoter region of Hsero_2339 cloned in pBSK | This work |
| pWS233 (pWS) | *Tc^R^*, suicide mobilizable vector,  *mob, sac^S^* | (Selbitschka et al., 1993) |
| pWS*fecA::lacZaacC1* | Hsero_1277 interrupted with *lacZaacC1* cloned in pWS | This work |
| pWS∆*fiu* | In frame deleted Hsero_3255 cloned in pWS | This work |
| pWS∆*ftr* | In frame deleted Hsero_2720 cloned in pWS | This work |
| pWS∆*cirA* | In frame deleted Hsero_2337 cloned in pWS | This work |
| pWS*pfrI::lacZaacC1* | Hsero_2338 interrupted with *lacZaacC1* cloned in pWS | This work |
| pAB2001 | *lacZaacC1* containing plasmid  *Amp^R^, lacZ-aacC1* | (Becker et al., 1995) |
| pA1∆*cirA* | In frame deleted Hsero_2337 cloned in pAB2001 | This work |
| pAB2002 | *lacZaacC1* containing plasmid  *Amp^R^, lacZ-aacC1* | (Becker et al., 1995) |
| pA2*pfrI::lacZaacC1* | Hsero_2338 interrupted with *lacZaacC1* cloned in pAB2002 | This work |
| pRK2013 | *Km^R^,* helper for triparental mating | (Ditta et al., 1980) |
| pCPP30 | *Tc^R^, lacZ* | (Huang et al., 1992) |
| pC*cirA* | Hsero_2337 cloned in pCPP30 under *plac* control | This work |
| pC*pfrI* | Hsero_2338 cloned in pCPP30 under *plac* control | This work |
| pSEVA237-C | *Km^R^,* CFP | (Martinez-Garcia et al., 2015) |
| p237*mbtH*pr | Promoter region of Hsero_2339 cloned upstream CFP in pSEVA237-C | This work |
|  |  |  |

*Nal^R^*: nalidixic acid resistance, *Amp^R^*: ampicilin resistance, *Tc^R^*: tetracyclin resistance, *Km^R^*: kanamycin resistance, *Str^R^*: streptomycin resistance.

References

Baldani, J.I., Baldani, B.V., Seldin, L., and Döbereiner, J. (1986). Characterization of Herbaspirillum seropedicae gen. Nov., a root-asociated nitrogen-fixing bacterium. *Int. J. Syst. Bacteriol* 36**,** 86-93.

Becker, A., Schmidt, M., Jager, W., and Puhler, A. (1995). New gentamicin-resistance and lacZ promoter-probe cassettes suitable for insertion mutagenesis and generation of transcriptional fusions. *Gene* 162(1)**,** 37-39.

Ditta, G., Stanfield, S., Corbin, D., and Helinski, D.R. (1980). Broad host range DNA cloning system for gram-negative bacteria: construction of a gene bank of Rhizobium meliloti. *Proceedings of the National Academy of Sciences of the United States of America* 77(12)**,** 7347-7351.

Huang, H.C., He, S.Y., Bauer, D.W., and Collmer, A. (1992). The Pseudomonas syringae pv. syringae 61 hrpH product, an envelope protein required for elicitation of the hypersensitive response in plants. *J Bacteriol* 174(21)**,** 6878-6885.

Martinez-Garcia, E., Aparicio, T., Goni-Moreno, A., Fraile, S., and de Lorenzo, V. (2015). SEVA 2.0: an update of the Standard European Vector Architecture for de-/re-construction of bacterial functionalities. *Nucleic Acids Res* 43(Database issue)**,** D1183-1189. doi: 10.1093/nar/gku1114.

Rosconi, F., Davyt, D., Martinez, V., Martinez, M., Abin-Carriquiry, J.A., Zane, H., et al. (2013). Identification and structural characterization of serobactins, a suite of lipopeptide siderophores produced by the grass endophyte Herbaspirillum seropedicae. *Environmental microbiology* 15(3)**,** 916-927. doi: 10.1111/1462-2920.12075.

Rosconi, F., Trovero, M.F., de Souza, E.M., and Fabiano, E. (2015). Serobactins mediated iron acquisition systems optimize competitive fitness of Herbaspirillum seropedicae inside rice plants. *Environmental microbiology*. doi: 10.1111/1462-2920.13202.

Selbitschka, W., Niemann, S., and Pühler, A. (1993). Construction of gene replacement vectors for Gram- bacteria using a genetically modified sacRB gene as a positve selection marker. *Applied microbiology and biotechnology* 38(5)**,** 615-618. doi: 10.1007/bf00182799.
