# Supplementary material for: Herbaspirillum seropedicae Differentially Expressed Genes in Response to Iron Availability
Source: Front Microbiol. 2018 Jul 3;9:1430. doi: 10.3389/fmicb.2018.01430 (PMC6037834; doi:10.3389/fmicb.2018.01430)
Supplement: Supplementary file 2 [file Table_2.DOCX]

**Table S2**. Primers designed for mutants construction and quantitative real-time PCR to amplify three genes probably involved in iron uptake mechanisms.

| **Gene** | **Primer** | **Sequence 5’-3’** |
| --- | --- | --- |
| Hsero_1277 | FecA1 | GAAGATCTAAGAAGCCGATGGACGCCGAG |
|  | FecA2 | GAAGATCTCGGGCCGCGGATCAGAATG |
| Hsero_3255 | FiuP1 | GGAATTCCCTGACCGGCAATTTCCACTTCTG |
|  | FiuP2 | GCCGCTGTCCACCTTGGTCGCGTGCTTGCGGCTTTTG |
|  | FiuP3 | GACCAAGGTGGACAGCGGC |
|  | FiuP4 | GCTCTAGAGCCGGCGCTCTCGAAACAAAGG |
| Hsero_2720 | FtrP1 | GGAATTCCCGGCAAGAAGGGCGGCTAC |
|  | FtrP2 | CAGGTCGTCATCAGGTCGTCAGACGACCTGTCCCATGAAACCCTTCC |
|  | FtrP3 | GTCTGACGACCTGATGACGACCTG |
|  | FtrP4 | GGAATTCCCGCGCTTTCCCAGAGATTGTTC |
| Hsero_2337 | CirA1 | AAATCTAGACTGCAGCTCGTTCGGCATACAAAG |
|  | CirA2 | AAACTCGAGCTGCAG GGACTTCGGCGATCTTGATG |
|  | CirA3 | CGTGACCTGCCGCCCCTG |
|  | CirA4 | GTCAGCAACCTCGCCAACCG |
| Hsero_2338 | PfrI1 | GGAAAACGCCGCCCAGGTCATC |
|  | PfrI2 | CACCGCATCGTCAGCATCAAAACTC |
| *p*Hsero_2339 | MbtHpr1 |  |
|  | MbtHpr2 |  |
| **Gene** | **qPCR primer** | **Sequence 5’-3’** |
| Hsero_1277 | Hsero1277For | CCGTTCTATGCCCGCACTAC ( 79 bp-amplicon) |
|  | Hsero1277Rev | TGGGTGGTCGACAGATTGAA |
| Hsero_2720 | Hsero2720For | GCGACGCCCAGACCTACTT (79 bp-amplicon) |
|  | Hsero2720Rev | GCGCATCCAGAACACCATCT |
| Hsero_0051 | Hsero0051For | TCCTGGTGCTGTTCTTCTTCATC (94 bp-amplicon) |
|  | Hsero0051Rev | CCCCACCGAGACCATCAG |

AGATCT : BglII restriction site

GAATTC: EcoRI restriction site

TCTAGA: XbaI restriction site

CTCGAG: XhoI restirction site
