# Supplementary material for: Herbaspirillum seropedicae Differentially Expressed Genes in Response to Iron Availability
Source: Front Microbiol. 2018 Jul 3;9:1430. doi: 10.3389/fmicb.2018.01430 (PMC6037834; doi:10.3389/fmicb.2018.01430)
Supplement: Supplementary file 4 [file Presentation_1.PDF]

Supplementary Figures

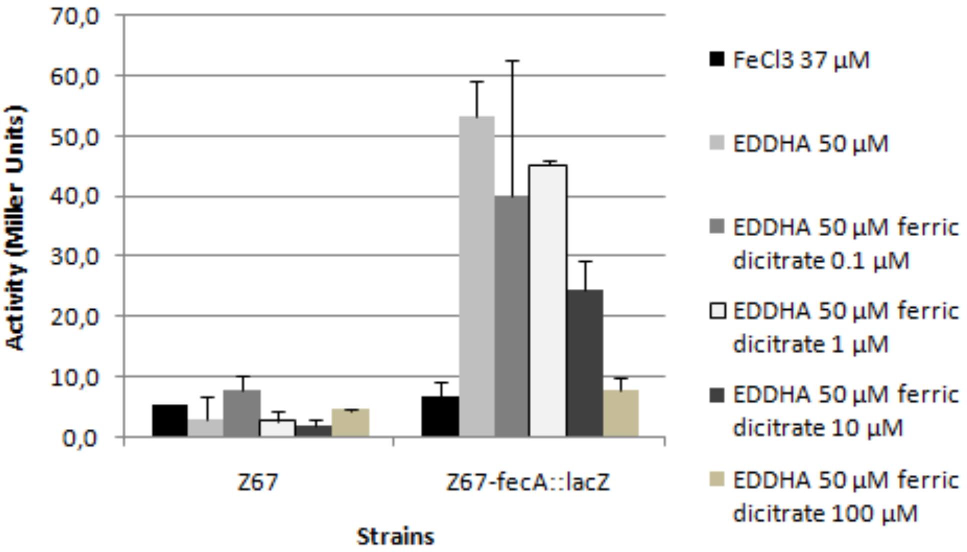

Figure S1. *fecA* expression assays measuring β-galactosidase activity in NFb-malate medium with iron (37 µM $FeCl_3$), without it (50 µM EDDHA), and with increasing concentrations of ferric dicitrate (0.1, 1, 10 and 100 µM) for *H. seropedicae*Z67 wild-type and *fecA* mutant. The experiment was done three times. Each column shows its error bar with standard deviation.

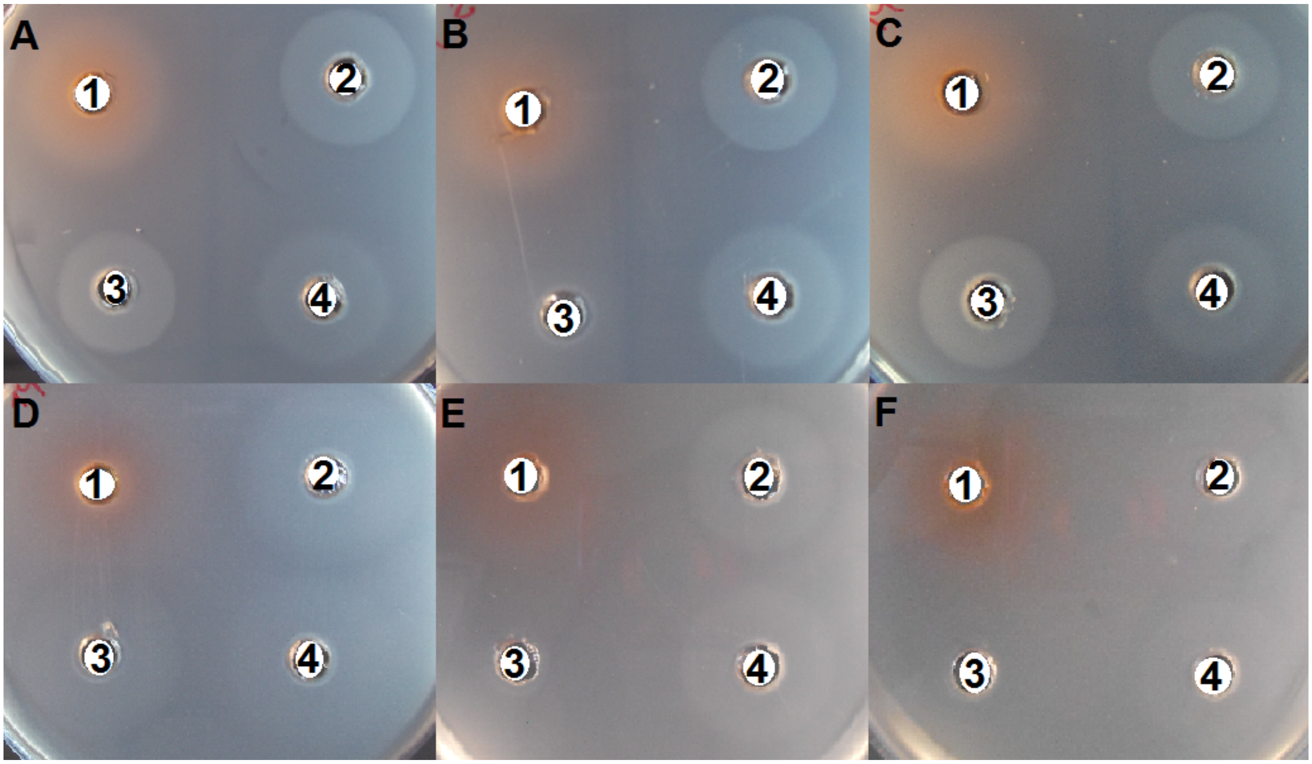

Figure S2. Utilization of different nutritional iron sources by the wild-type (A), *sbtR* (B), *fecA* (C), *fiu* (D), *sbtI/sbtR/fiu* (E) and *fecA/sbtR/fiu* (F) mutant strains. Bioassay experiments were done in TY medium with 400 µM EDDHA. Iron sources tested were: 37 mM $FeCl_3$ (1), 0.5 mM ferrichrome (2), 300 nM ferric serobactin (3) and 2 mM ferric dicitrate (4).

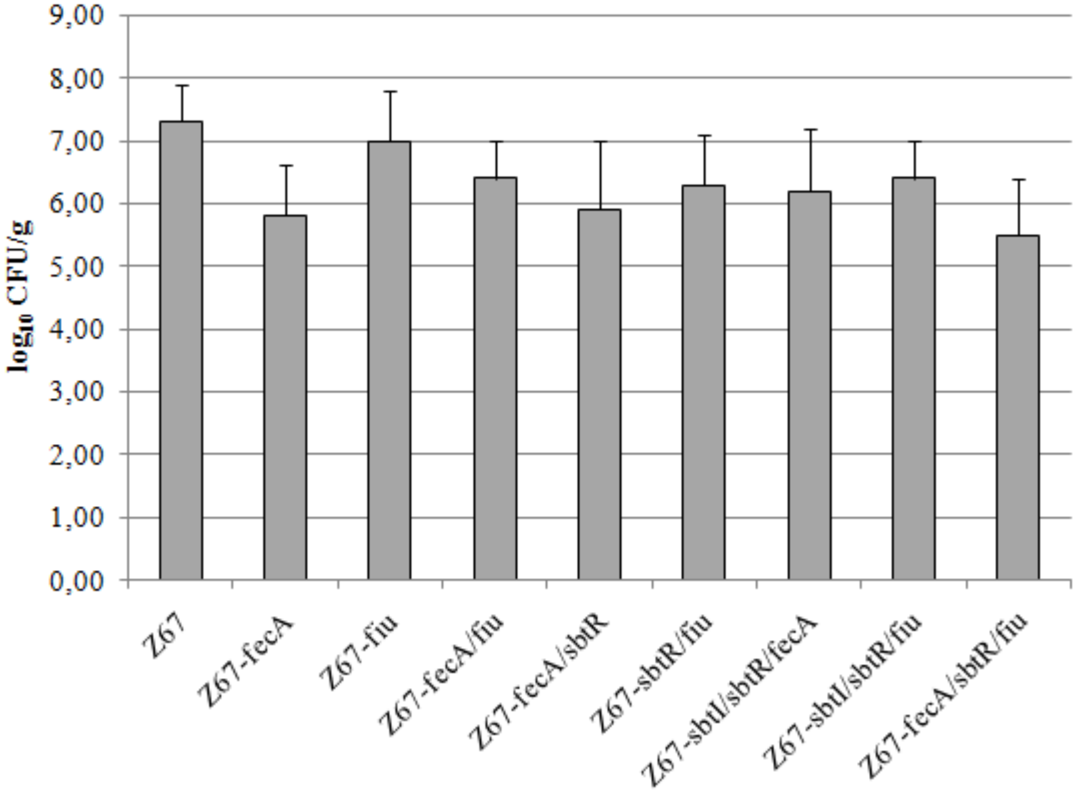

Figure S3. Rice colonization assays by different strains. Recovered bacteria 8 days post-inoculation, expressed as $\log_{10}$ CFU/g of plant-aerial part. Each column presents a bar with standard deviation. Statistical analysis did not show significant differences.

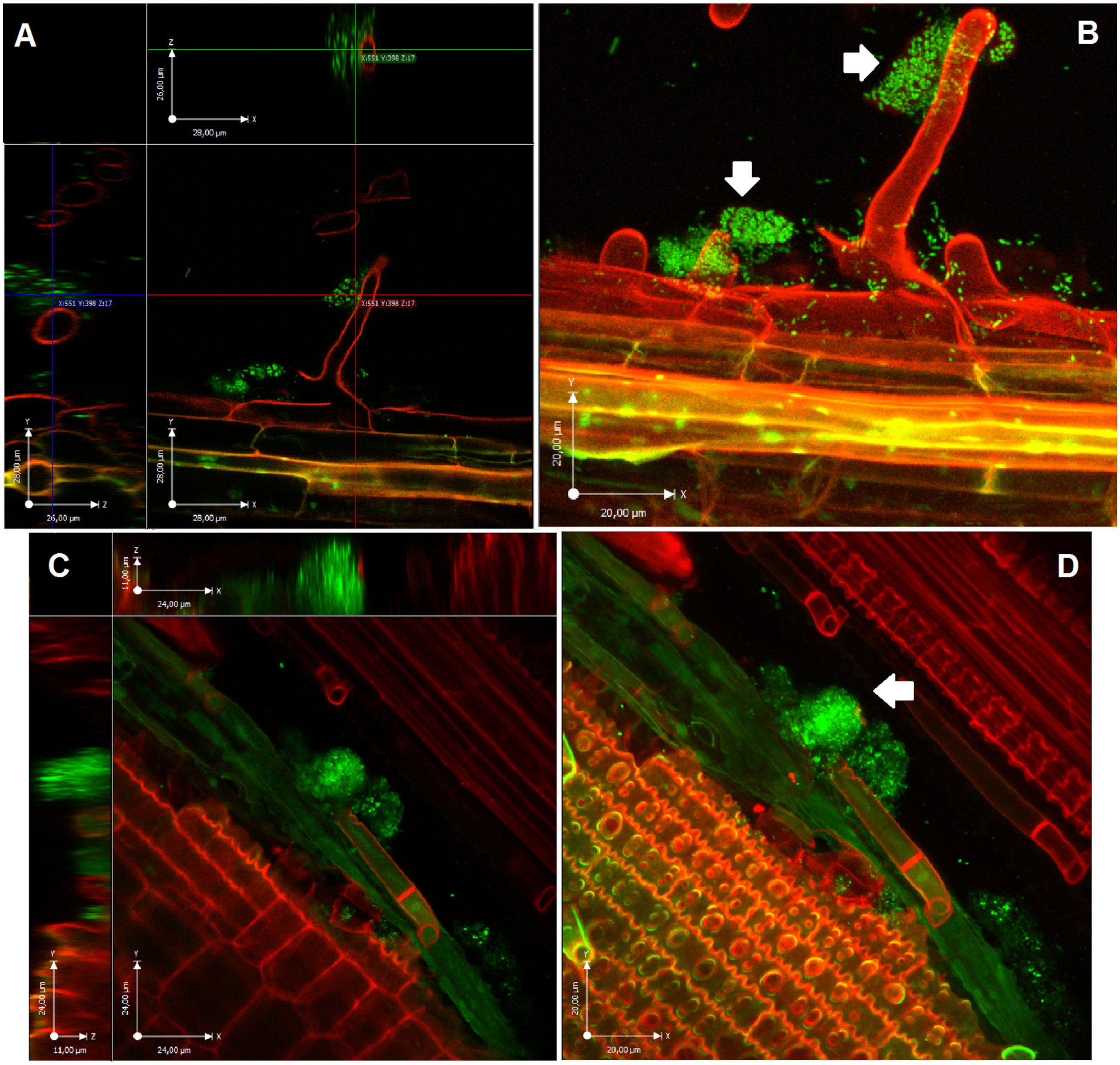

Figure S4. Biofilm formation by *H. seropedicae* Z67 in rice root (A and B) and by *sbtI/sbtR* mutant strain in plant-aerial part of rice (C and D). Optical sections (A and C) and extended focus (B and D). The auto-fluorescense is seen in red, and in green it is also seen the specific Syto9 mark. Arrows point biofilm agglomerates.
